# Are tuberculosis patients adherent to prescribed treatments in China? Results of a prospective cohort study

**DOI:** 10.1186/s40249-016-0134-9

**Published:** 2016-05-05

**Authors:** Xun Lei, Ke Huang, Qin Liu, Yong-Feng Jie, Sheng-Lan Tang

**Affiliations:** China Effective Health Care Network, Research Center for Medicine and Social Development, Innovation Center for Social Risk Governance in Health, School of Public Health and Management, Chongqing Medical University, Chongqing, China; Public Health Clinical Center of Chengdu, Chengdu, China; Changhang Hospital of Chongqing, Chongqing, China; Duke University, Durham, NC USA

**Keywords:** Tuberculosis, Adherence, Prospective cohort study, China

## Abstract

**Background:**

Tuberculosis (TB) patients face numerous difficulties adhering to the long-term, rigorous TB treatment regimen. Findings on TB patients’ treatment adherence vary across existing literature and official reports. The present study attempted to determine the actual treatment adherence of new TB patients and to identify factors leading to non-adherence.

**Methods:**

A prospective cohort of 481 newly confirmed TB patients from three counties in western China were enrolled during June to December 2012 and was followed until June 2013. Patients who missed at least one dose of drugs or one follow-up re-examination during the treatment course were deemed as non-adherent. Influencing factors were identified using a logistic regression model.

**Results:**

A total of 173 (36.0 %) patients experienced non-adherence and the loss to follow-up cases reached 136 (28.2 %). Only 13.9 % of patients took drugs under direct observation, and 60.5 % of patients were supervised by phone calls. Factor analyses suggested that patients who were observed by family members (*OR*:5.54, 95 % *CI*:2.87–10.69) and paying monthly service expenses above 450 RMB (*OR*:2.08, 95 % *CI*:1.35–3.19) were more likely to be non-adherent, while supervision by home visit (*OR*:0.06, 95 % *CI*:0.01–0.28) and phone calls (*OR*:0.27, 95 % *CI*:0.17–0.44) were protective factors.

**Conclusions:**

Despite recent efforts, a large proportion of newly confirmed TB patients could not adhere to standard TB treatment, and patients’ lost to follow-up was still a serious problem. Poor treatment supervision and heavy financial burden might be the main causes for non-adherence. More needs to be done to enhance treatment supervision policies and financial supports to both health providers and TB patients.

**Electronic supplementary material:**

The online version of this article (doi:10.1186/s40249-016-0134-9) contains supplementary material, which is available to authorized users.

## Multilingual abstracts

Please see Additional file 1 for translations of the abstract into the six official working languages of the United Nations.

## Background

Tuberculosis (TB) remains a major threat to global public health, with an estimated 9.6 million new cases and 1.5 million deaths worldwide in 2014 [1]. Standard anti-TB treatment requires patients to take a complex combination of drugs every other day, lasting for 6 months for new patients and 8 months for retreatment patients [2]. Such long-term, strict regimens is challenging for TB patients who may not adhere to their prescribed treatment due to treatment interruption or drop-out [3]. Non-adherence is the most common cause of treatment failure and disease relapse, which can lead to prolonged infection, transmission, drug resistance and mortality [3, 4]. Directly observed therapy (DOT), which requires TB patients to take medicine under the direct observation of health workers or family members, was the key element of the DOTS strategy recommended by the World Health Organization (WHO) and was shown effective in improving adherence to anti-TB treatment [5]. However, DOT coverage rates were still low and made little improvement on treatment adherence in some resource-limited countries [6, 7].

Even with over 300 million USD funding from the World Bank, Global Found and other international agencies, and with counterpart funding from the Chinese government each year [8, 9], TB control remains poor. China still reports the world’s second highest TB prevalence. As WHO reported, 0.8 million Chinese people were estimated to have fallen ill with TB in 2014 and 2 % of those new TB cases were HIV-positive [1]. According to the fifth National TB Epidemiological Survey in 2010, the country’s active TB prevalence had “almost no change” during the past decade (466/100 000 in 2000 vs. 459/100 000 in 2010) [10]. Non-adherence to TB treatment was considered an important cause of the gap between high financial inputs and poor performance in TB control [11].

The national TB survey also reported that 25.3 % of TB patients missed more than one dose of anti-TB drugs [10]. Existing studies undertaken in both western and eastern Chinese provinces also showed the non-adherence rate to range from 9.4 to 25.9 %. Factors affecting patient adherence includes education level, adverse effects of treatment, financial status and treatment supervision [12–14]. Most evidence come from retrospective surveys or facility-based routine reporting records, and findings vary widely across different sources [12]. We therefore aim to estimate the current TB treatment adherence in western China and identify its influencing factors using a prospective cohort study.

## Methods

The study was carried out in Chongqing, China. And the study followed the STROBE guidelines for a cohort study.

### Chongqing’s TB control and routine strategies to ensure TB treatment adherence

Chongqing is located in a mountainous area in western China, with a population of 30 million. Chongqing has been regarded as “miniature China” because its geographic feature, urban–rural distribution and socio-economic profile were close to the national [15]. According to China’s Fifth Tuberculosis Epidemiological Sampling Survey in 2010, the active TB prevalence in Chongqing was 651/100 000 and 70 % of the TB cases were in the rural areas. As reported by the local infectious diseases information management system, the notification rate of new and relapse pulmonary TB cases was 83.3/100 000 and the detection rate of TB/HIV co-infection was 1.1 % based on patient screening in 2013. The treatment completion rate of new and relapse pulmonary TB patients reached 90 %, which was comparable with the national average [16, 17].

The TB control system in China is a semi-vertical system including TB facilities at four levels: national, provincial, municipal, and county. Chongqing’s TB control follows the principles of China’s NTP [18]. In each county of Chongqing, the TB control activities are mainly performed by county TB dispensaries or the hospitals designated by the local health authorities for TB care (called “designated hospital” for short), under the supervision of provincial TB dispensary and Health Bureau. Suspect TB cases detected in general hospitals/clinics are required to be referred to TB dispensaries (affiliated with Center for Disease Control in some counties) or designated hospitals for treatment and management. According to the NTP guidelines [18], both new smear-positive and smear-negative pulmonary TB patients were treated with chemotherapy regimen 2H_3_R_3_Z_3_E_3_/4H_3_R_3_ (2-month intensive phase with Isoniazid, Rifampicin, Pyrazinamide and Ethambutol every other day, and 4-month continuation phase with Isoniazid and Rifampicin every other day), while a new regimen named fixed-dose combination (FDC, a daily formula combining appropriate doses of Isoniazid, Rifampicin, Pyrazinamide and Ethambutol) is recently recommended for TB treatment in Chongqing. Newly confirmed TB cases should be provided with free therapy and patients should take each dose of anti-TB drug under the direct observation by health workers (usually community or village doctors) during the 6-month treatment course no matter how far they reside from the health facility (strict DOT implementation). Some smear-negative cases are allowed to receive DOT during the first 2-month intensive phase, and then regularly collect TB drugs at dispensaries/designated hospitals (usually once a month) during the continuation phase. Health workers are responsible for reminding the patients about their next scheduled appointment at the time of medicine collection, and performing regular home visits for patient management. In addition, family members of TB patients are strongly advised to help patients finish regular treatment; therefore, both patients and family members should be educated by health workers on TB and its treatment (standard regimen, treatment duration, possible adverse effects, disease evolution, household prevention and consequences of non-adherence).

### Sampling and population

The sampling process was performed in three steps: In step 1, all 39 counties of Chongqing were stratified into three groups (developed urban area, rural–urban connective area and poor rural area). In step 2, one county from each group was selected as the study site depending on our partnership with local health authorities. The three selected counties were CC (representing urban counties), SS (representing urban–rural counties) and YY (representing rural counties). In step 3, all the pulmonary TB (PTB) patients newly-diagnosed by chest X-ray and sputum smear (both positive and negative ones), registered at the outpatient department of the TB dispensaries and designated hospitals between 1 June and 31 December 2012 were enrolled into the cohort. It is noteworthy that in Chongqing only patients with severe complications should be hospitalized in the specialist TB hospital, and then transferred to local TB dispensaries or designated hospitals for subsequent outpatient treatment and management. Thus, study only recruited outpatients from the TB dispensaries and designated hospitals.

### Data collection

Patients were recruited to the cohort between June and December 2012, and were followed for 6 months, as the recommended treatment course for a new TB case is 6 months. The observation period ended in June 2013. Data collection was undertaken by a team of trained researchers at the beginning and end of each month of the treatment period. Data from both smear-positive and smear-negative patients were obtained using self-designed questionnaires, covering information on patient’s demographic characteristics, type of TB diagnosis, treatment regimen, medication supervision, drug-taking pattern, hospital appointments, adverse effects, treatment outcomes, costs. In addition to conducting patient survey, patients’ treatment cards were checked to confirm the medication collection and hospital visits. Non-adherence was defined as missing at least one dose of drugs or one follow-up appointment during the treatment course, including interrupters, defaulters and lost to follow-up cases (detailed definitions see Table 1) [5, 6, 12–14, 19].

**Table 1:** Definitions for this study

1. Treatment adherence was defined as regular medication intake and attending all follow-up medical appointments according to the guidelines. On the contrary, non-adherence referred to missing at least one dose of drugs or one follow-up appointment during the treatment course. 2. Treatment success referred to the completion of the full course of standard treatment and to the curing (become smear-negative) of newly registered smear-positive cases. For patients who started treatment as smear-negative cases, treatment success referred to the completion of the standard treatment course (including the interrupting cases). 3. Interrupter was defined as a patient who pauses treatment for more than three doses or for more than a week but less than eight weeks (eventually returning for treatment). 4. Defaulter was defined as the interruption of treatment for more than eight consecutive weeks. The default case usually needs re-treatment. 5. Lost to follow-up meant patients who dropped out the cohort because of loss of contact or any other reasons.	

### Data analysis

Survey data were double entered using EpiData 3.1 software, and analyzed using SPSS 19.0 (Chicago, USA). Categorical data was described using frequencies and percentages, while continuous data such as age and income, were converted to categorical variables using the quartile or median as cutoff points. The associations between TB adherence and independent variables were firstly estimated using unconditional logistic regression, and assessed by odds ratio (*OR*) with 95 % confidence intervals (*CI*s). Then the variables with *P*-value less than or close to 0.10 in the univariate analysis were included in a multivariate logistic regression model (α = 0.05, β = 0.1), where all independent variables were dummy-coded. Results were statistically significant if the *P*-value <0.05. The model was statistically significant in the model coefficient test (*P* < 0.05) and reached a good fit in Hosmer & Lameshow test (*P* > 0.20). Significance of the variable blocks were assessed via the likelihood ratio test (G − 2Log likelihood = 223.57, *P* < 0.05).

## Results

A total of 481 newly confirmed TB cases, including 156 (32.4 %) smear-positive cases and 325 (67.6 %) smear-negative cases, were enrolled and followed in the cohort, and no drug resistant case or TB/HIV co-infection were detected during the study period. 326 (67.8 %) of enrolled patients were male, and the age ranged from 14 to 89 years with median age being 39 years. 422 (87.7 %) patients were long-term residents, and 387 (80.5 %) were covered by at least one type of health insurance scheme. The average annual income of participants was 12 758 RMB and only 78 (16.2 %) patients had an income of over 18 000 RMB (Table 2).

**Table 2:** Univariate analysis of association between influencing factors and treatment adherence (*n* = 481)

Variables	Total n	Adherent		*OR* (95 % *CI*)	*P* value
		Yes, n (%)	No, n (%)		
Gender					
Male	326	199 (61.0)	127 (39.0)	1.00	
Female	155	109 (70.3)	46 (29.7)	0.66 (0.44-0.99)	**0.047** ^f^
Age (in years)					
≤18	43	23 (53.5)	20 (46.5)	1.00	
19–49	297	199 (67.1)	98 (32.9)	0.57 (0.30-1.08)	0.820
≥50	141	86 (61.0)	55 (39.0)	0.74 (0.37-1.46)	0.381
Occupation^a^					
Farmer	86	57 (66.3)	29 (33.7)	1.00	
Employed worker	166	105 (63.3)	61 (36.8)	1.14 (0.66-1.97)	0.635
Student	65	40 (61.5)	25 (38.5)	1.31 (0.67-2.56)	0.427
Laid-off or unemployed	85	57 (67.1)	28 (32.9)	0.97 (0.51-1.82)	0.914
Others	79	49 (62.0)	30 (38.0)	1.20 (0.64-2.28)	0.569
Educational level					
Primary school or below	120	69 (57.5)	51 (42.5)	1.00	
Junior middle school	154	102 (66.2)	52 (33.8)	0.69 (0.42-1.13)	0.139
High school and above	207	137 (66.2)	70 (33.8)	0.69 (0.44-1.10)	0.127
Marital status					
Married	360	230 (63.9)	130 (36.1)	1.00	
Unmarried	106	67 (57.6)	39 (42.5)	1.03 (0.66-1.61)	0.898
Divorced/loss of spouse	15	11 (73.3)	4 (26.7)	0.64 (0.20-2.06)	0.455
Annual per capita income (RMB)^b^					
≤9000	198	118 (59.6)	80 (40.4)	1.00	
9000-18000	193	128 (66.3)	65 (33.7)	0.75 (0.50–1.13)	0.169
≥18000	78	51 (65.4)	27 (34.6)	0.78 (0.45–1.35)	0.374
Study site					
CC	101	65 (64.4)	36 (35.6)	1.00	
SS	185	128 (69.2)	57 (30.8)	0.79 (0.48–1.31)	0.146
YY	195	115 (59.0)	80 (41.0)	1.26 (0.76–2.07)	0.369
Residence status^c^					
Local residents	422	275 (65.2)	147 (34.8)	1.00	
Migrant workers	59	33 (55.9)	26 (44.1)	1.47 (0.85–2.56)	**0.106**
Time spending to TB care institution					
≤ One hour	284	191 (67.3)	93 (32.8)	1.00	
> One hour	197	117 (59.4)	80 (40.6)	1.43(0.98–2.09)	**0.060**
Health insurance					
Yes	387	241 (62.3)	146 (37.7)	1.00	
No	94	67 (71.3)	27 (28.7)	0.67 (0.41–1.09)	**0.103**
Pulmonary TB type					
Smear-positive	156	105 (67.3)	51 (32.7)	1.00	
Smear-negative	325	203 (62.5)	122 (37.5)	1.24 (0.83–1.85)	0.300
TB-related education by physicians					
Yes	280	188 (67.1)	92 (32.9)	1.00	
No	201	120 (62.7)	81 (37.3)	1.38 (0.952.01)	**0.093**
Treatment observation					
Self-administrated	414	278 (67.2)	136 (32.9)	1.00	
Family members	55	18 (32.7)	37 (67.3)	4.20 (2.31–7.65)	**<0.001**
Primary health workers	12	11 (91.7)	1 (8.3)	0.67 (0.63–0.72)	**<0.001**
Regular supervision contact					
No supervision contact	152	67 (44.1)	85 (55.9)	1.00	
Home visit	38	36 (94.7)	2 (5.3)	0.44 (0.01–0.19)	**<0.001**
Telephone call	291	205 (70.5)	86 (29.6)	0.33 (0.22–0.50)	**<0.001**
Adverse effects^d^					
Yes	123	80 (65.0)	43 (35.0)	1.00	
No	358	228 (63.7)	130 (36.3)	1.06 (0.69–1.63)	0.787
Monthly treatment cost^e^ (CNY)					
≤450	246	172 (69.9)	74 (30.1)	1.00	
>450	235	136 (57.9)	99 (42.1)	1.69 (1.16–2.46)	**0.006**

^a^In the occupation category, employed workers include factory worker, building worker, food service staff, driver, administrative sector staff and teacher; others include individual peddler, freelance and retired staff

^b^Annual per capita income refers to ‘Chongqing Statistic Yearbook’ [Chongqing statistical yearbook (in Chinese). Chongqing: China Statistics Bureau Press; 2013], the annual income levels of urban and rural areas were categorized into middle-income (CNY 18000) and low-income (CNY 9000). A total of 12 patients did not fill in this blank

^c^Local residents in our study mean the patients have lived and worked in the study sites for at least 12 months; migrant workers refer to the patients temporarily living in the study sites and receiving treatment but may move along with floating work at any time

^d^Adverse effects include nausea or vomiting, hands or feet numbness, dizziness, headaches, insomnia, skin rash, etc

^e^Treatment cost include direct expenditure on self-paid TB drugs, liver protecting drugs, cough remission drugs, traveling, accommodation and nourishment. 450 RMB is the median cost

^f^Variables with a *P*-value less than or close to 0.10 in the univariate analysis were included in the multivariate logistic regression analysis

**Fig. 1:**
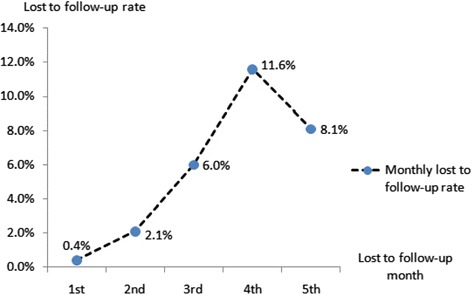
Monthly lost to follow-up rates of cohort participants. Monthly lost to follow-up rates were calculated as the number of patients quitting on which month of the treatment divided by the overall 481 participants

During the study period, 308 participants (64.0 %) took medications regularly as prescribed and completed re-examinations as scheduled. Among adherent patients, the treatment success rate reached 82.5 % (including 51 smear-positive cases that became negative and 203 treatment completion cases). Of 173 non-adherence patient, 136 were lost to follow-up, including 110 cases that lost contact and 26 that left for work, totally accounted for 28.2 % of all participants. The monthly loss to follow-up rates increased each month with a peak (11.6 %) in the fourth month of treatment (Fig 1). In addition, the rest 37 non-adherent cases (7.7 %) took drugs intermittently. No TB case died during the study period.

280 cases (58.2 %) received TB related education before or during their chemotherapy, and only 67 (13.9 %) patients said that they were observed when taking their medication, including twelve (2.4 %) observed by primary health workers and 55 (11.5 %) by family members. The rest 414 patients (86.1 %) took drugs without another person observing. Most patients (60.5 %) were inquired about and reminded of drug taking and re-examination through telephone calls during the treatment course. Few patients (7.9 %) were visited by health workers and 152 cases (31.6 %) reported that they never received any supervision contact (Table 2).

Univariate logistic analysis indicated that non-adherence was associated with gender male (*OR*:0.66, 95 % *CI*:0.44–0.99), family member observation or monthly medical service expenses above 450 RMB (*OR*:4.20, 95 % *CI*:2.31–7.65). Protective factors included health worker observation (*OR*:0.67, 95 % *CI*:0.63–0.72), home visit (*OR*:0.44, 95 % *CI*:0.01–0.19) and telephone calls supervision (*OR*:0.33, 95 % *CI*:0.22–0.50) (Table 2). Multivariate logistic regression (Table 3) showed that patients who were observed by family members (*OR*:5.54, 95 % *CI*:2.87–10.69) and with monthly treatment expenses more than 450 RMB (*OR*:2.08, 95 % *CI*:1.35–3.19) were more likely to be non-adherent, while home visit supervision (*OR*:0.06, 95 % *CI*:0.01–0.28) and telephone calls (*OR*:0.27, 95 % *CI*:0.17–0.44) were associated with better treatment adherence (Table 4).

**Table 3:** Dummy variables coding of variable values for multivariate logistic regression

	Indicator	Category
Dependent variable	TB treatment adherence	0 = adherence; 1 = non-adherence
Independent variable	Gender	0 = male; 1 = female
	Residence status	0 = migrant workers; 1 = local residents
	Time spending to TB care institution	0 = ≤ one hour; 1= > one hour
	Health insurance	0 = yes; 1 = no
	TB-related education by physicians	0 = yes; 1 = no
	Treatment observation	0 = self-administrated; 1 = family members; 2 = primary health workers
	Regular supervision contact	0 = no supervision contact; 1 = home visit; 2 = telephone call
	Monthly treatment cost	0 = ≤450; 1= >450

**Table 4:** Multivariate logistic regression on association between selected factors and treatment non-adherence

Variables	*OR*	*OR* 95 % *CI*		*P* value
		Lower	Upper	
Gender (compared with ‘male’)				
Female	0.69	0.44	1.09	0.109
Residence status (compared with ‘migrant workers’)				
Local residents	0.75	0.39	1.42	0.375
Time spending to TB care institution (compared with ‘≤ One hour’)				
> One hour	1.13	0.65	1.94	0.671
Health insurance (compared with ‘no’)				
Yes	0.62	0.34	1.13	0.116
TB-related education by physicians (compared with ‘yes’)				
No	1.02	0.60	1.74	0.934
Treatment observation (compared with ‘self-administrated’)				
Family members	5.54	2.87	10.69	**<0.001** ^a^
Primary health workers	1.18	0.92	15.18	0.899
Regular supervision contact (compared with ‘no supervision contact’)				
Home visit	0.06	0.01	0.28	**<0.001**
Telephone call	0.27	0.17	0.44	**<0.001**
Monthly treatment cost (compared with ‘≤450’)				
>450	2.08	1.35	3.19	**<0.001**

^a^Results were statistically significant when the *P*-value less than 0.05

## Discussion

Adherence to TB treatment is crucial for effective TB control. However, current long-term anti-TB treatment regimen might cause patient non-adherence [19]. In our study, only 64.0 % of participants were able to adhere to the defined regimens and completed the whole treatment course, which was lower than the reported adherence rates in Jiangsu Province (87.8 %), and Shandong Province (84.0 %), and the average level of other mountainous provinces (79.0 %). Furthermore, our study found lower adherence rate compared with previous survey data from new TB patients in Chongqing Province (74.1 %) [6, 12–14], however, it was probably close to the real situation of treatment adherence for newly confirmed TB cases given the prospective cohort data. Several possible reasons for the low adherence rate observed in our study are: 1) Definitions of non-adherence for TB treatment varied across studies. There is yet no empirical evidence or consensus to the best definition of non-adherence to TB treatment, although WHO recommended a definition of non-adherence based on the quantity and timing of missed medication or hospital appointments. In our study patients who missed at least one dose of drug or one follow-up appointment were deemed as non-adherent, which was relatively rigorous. Some studies have defined it as patients who missed 10 % or more of the total prescribed dose of TB drugs and some defined it as discontinue medication for 6 days [12, 13]. 2) Officially reported data on TB treatment adherence might be sometimes over-estimated or over-reported. 3) Adherence rate might have indeed declined in recent years. 4) A large number of lost to follow-up participants, who were sometimes excluded in retrospective surveys and analyses, were also counted as non-adherent cases in our study because they were unlikely to attend medical appointments for medicine collection or re-examinations. Such a high lost to follow-up rate (28.2 %) has highlighted the importance of maintaining long-term follow-up with TB patients. In addition, the dropping out cases of treatment due to relocation for working suggested that existing internet-based surveillance systems needs strengthening for cross-regional supervision and management [20]. Moreover, some compulsory measures, such as wage deduction or working prohibition of workers who do not regularly take TB drugs, might be effective in the workplace [21]. Having said that, association between treatment adherence and possible influencing factors could also be detected based on available data regardless of the number and time of participants quitting the cohort because all the lost to follow-up cases were viewed as non-adherent in factor analyses.

Observing patients’ taking medication is crucial in TB therapy. China’s national TB control guidelines are consistent with WHO’s DOTS strategy, in which the trained agent, including health worker, community volunteer or family member, are recommended to directly supervise patients’ medication intake [18, 19]. An optimistic report on DOTS coverage stated that all TB patients had been provided with DOTS in China until 2005 [18]. However, the National TB Epidemiological Survey revealed that only 64.2 % of detected TB patients were under the case management of TB dispensaries/designated hospitals and less than 75 % of them regularly took medication [10]. In our study, only those patient observed by health works and family members (13.9 %) met the DOT requirements. Consistent with our findings, DOT coverage was found low in rural areas (8.4 %), mountainous areas (28.8 %) and some eastern coastal provinces (21.0 %) [13, 14, 22]. All these evidence suggested that DOT were not well implemented in China and some researchers have argued that DOT may not be a practical policy option [12]. In fact, Chinese primary health care providers, including urban community doctors and rural village doctors, were important players in the national TB control system and were responsible for home visits and supervising drug taking [23]. However, a number of patients lived in rural or mountain areas without convenient transportation, and require long travel time for home visits. These factors might create a barrier for proper supervision by health workers [12, 14]. Moreover, our previous in-depth interviews revealed that primary health care staff were paid 100 RMB for the full-course of direct supervision in some counties of Chongqing, which was far from enough for such demanding work, and was even insufficient to cover the doctors’ traveling expenses [24]. Therefore, higher financial incentives may help encourage health staff to practice better management and supervision of TB patients, at least during the intensive phase (first 2 months) of TB treatment [25]. Family members were also expected to be a possible alternative for patient supervision. Prior studies confirmed their positive effect on treatment adherence [20, 22]. But our findings, on the contrary, suggested that family observation was probably a risk factor for poor treatment adherence. In practice, where family observation was allowed, family members might not be strict or responsible due to their lack of training by the health care system. Patients were often merely handed medicines and have their spouse watch them swallow the pills [26]. In addition, definition of family observation should be clarified because some patients put ambiguous understanding on the duty of family observation. Sometimes, family members might just “happened to be in the room” and occasionally remind patients of taking drugs without actually regularly observing the medication intake [12]. Further, family members should receive TB related training in order to supervise patients. Feasible external intervention technology like the Short Message Service or smart phone applications might be helpful for reminding and guiding both patients and family members [27, 28].

Given the barriers of treatment supervision by health workers and family members, regular supervision contact between doctors and TB patients is essential. In our study, factor analysis suggested that supervision contact via home visits and phone calls could improve treatment adherence. Our study indicated that 60.5 % of patients reported that they had received non-scheduled phone calls from health staff to remind them of medications and hospital appointments schedule and to pick up TB medicine before it runs out, on the other hand, home visits for drug delivery and health services were rarely seen. In fact, China’s TB guidelines recommended that new pulmonary TB patients who were provided with free anti-TB drugs should be observed by health workers during the whole treatment course [18]. However, direct observation by health workers were hardly implemented because most TB facilities in Chongqing were understaffed [24], and telephone calls from health workers became the most commonly adopted method for patient management. Nevertheless, the time of telephone contact might differ across TB facilities due to lack of standard. In addition, health workers had little incentive for contacting patients, thus, they may be less motivated to perform regular supervision calls, which would increase their workload. Therefore, more specific and stronger policies are needed to improve and standardize phone call supervision. In addition, patients in the fourth or fifth month of their treatment course had a high loss to follow-up rate. It’s unclear whether this is specific to this study or is a common problem since previous publications rarely discussed on this issue.. One possible reason of loss to follow-up later in the treatment was because patients had symptom relief and perceived further treatment as unnecessary [11]. This suggest, in later part of the treatment, more routine contact and reminders are necessary to inform them of the importance of treatment compliance and educate patients on the negative consequences of treatment termination including the high possibility of TB relapse and drug resistance.

High treatment costs remain a serious problem in TB control [29]. The treatment cost was found to contribute to non-adherence in our study. China’s national TB programme (NTP) guidelines requires free provision of first-line TB treatment for 6 months, two X-ray examinations and all sputum smear examinations [2]. However, this so-called “free TB service policy” did not cover the expenses of second-line TB drugs, drugs related to respiratory symptoms, liver protection, or adverse effects. In addition, patients have costs related to transportation and accommodation. The perverse incentives in many TB facilities or hospitals had significantly increased patients’ financial burden as well [12, 13, 30]. Most TB health workers in Chongqing had low remuneration for providing medical service, and the TB department was viewed as less profitable [24], therefore, TB doctors may over-prescribe drugs and tests. The profit-seeking behavior was an important cause of financial burden for TB patients and some prescribed treatments or tests are not necessary [30, 31]. To tackle this issue, incentives should be reformed to motivate TB health providers to provide appropriate treatment. Meanwhile, monitoring mechanisms, regulations and laws should be established to regulate provider behaviors. In addition, our study showed that the average annual income of TB patients was 12 758 RMB, lower than the average of all Chongqing residents (24 565 RMB) and national average (22 968 RMB) [15]. At present, each TB outpatient in Chongqing could receive an annual reimbursement of 1 000 RMB [32], which was not enough to cover treatment expenses as most patients spent hundreds to thousands of RMB per month on TB treatment. More subsidy or reimbursement is needed to reduce patients’ financial burden.

Gender difference in treatment adherence was detected in univariate analysis, which was consistent with a previous study [33]. Interviews with male TB patients (unpublished) revealed that male patients were more likely to be non-adherent to prescribed regimen because of their unhealthy lifestyle and overconfidence in their health status. Although newly diagnosed TB patients should receive TB-related education by health workers [18], only 58.2 % of participants were actually educated by doctors before or during TB treatment in our study, which highlights the need for better patient education because poor TB-related knowledge was often associated with non-adherence [6, 20].

Several methodology issues need to be discussed. Firstly, the design of the present study is a prospective cohort study, for which we could collect data of good quality and make relatively exact estimates on treatment history and outcomes, possible explanatory factors. The design also minimizes patients’ recall bias and avoids over- or under-estimation in official reports or retrospective surveys. Secondly, when analyzing influencing factors, we actually performed grouping according to outcome variable (adherence or not) as it was impossible to group based on diverse individual causal factors, and given that different types of variables were involved, multivariate logistic regression was used and odds ratios (*OR*s) were directly computed. *OR* is a commonly used indicator to detect the association between a disease and exposure factors in epidemiology research; however, it might sometimes overstate the effect size and is more difficult to interpret than the relative risk (RR) which indicates a ratio of two rates [34, 35]. The study also has several limitations. Firstly, non-randomized sampling method might give rise to selection bias. Secondly, patients lost to follow-up were counted as non-adherent cases in our study based on WHO’s definition, since those patients were likely to skip medical appointments. Consequently, it was difficult to measure the treatment success (cure and completion) as well as regular medication pattern of those lost cases.

## Conclusion

Compared to previous retrospective studies on TB adherence, our prospective cohort study revealed relatively high non-adherence rate among new pulmonary TB patients. One third of participants experienced non-adherence, and patients’ lost to follow-up was not uncommon. Poor treatment supervision and heavy financial burden were identified as the main causes of non-adherence. Policies are suggested to be developed to regulate and strengthen the treatment management of TB patients, and feasible technical methods such as electronic/mobile health interventions might be introduced to ensure regular medication intake and follow-up visits. Moreover, better financial incentives for health workers are needed to improve patients supervision, and more financing support, such as promote the reimbursement level of treatment, are desirable to reduce TB patients’ financial burden.

## Ethics approval and consent participate

Ethical approval was granted by the Medical Ethics Review Committee of Chongqing Medical University. All TB patients were asked about their willingness to participate in the study and written informed consent was obtained before the survey.

## Additional file

Additional file 1: Multilingual abstracts in the six official working languages of the United Nations. (PDF 304 kb)

## Abbreviations

CI: confidence interval
DOT: directly observed therapy
DOTS: directly observed treatment, short-course
FDC: fixed-dose combination
NTP: national TB programme
OR: odds ratio
PTB: pulmonary TB
TB: Tuberculosis
WHO: World Health Organization
