# Supplementary material for: Are tuberculosis patients adherent to prescribed treatments in China? Results of a prospective cohort study
Source: Infect Dis Poverty. 2016 May 5;5:38. doi: 10.1186/s40249-016-0134-9 (PMC4857377; doi:10.1186/s40249-016-0134-9)
Supplement: Additional file 1: — Multilingual abstracts in the six official working languages of the United Nations. (PDF 304 kb) [file 40249_2016_134_MOESM1_ESM.pdf]

Translation of the abstract into the five official working languages of the United Nations

هل يلتزم مرضى السل بالعلاج في الصين؟ نتائج دراسة الأتراب المحتملين

شون لي، كه هوانج، كين ليو، يونج-فينج جي، شينج-لان تانج

ملخص

معلومات عامة: يواجه مرضى السل (TB) العديد من الصعوبات للالتزام بالنظام العلاجي الصارم طويل الأجل. تتفاوت النتائج الواردة بالمنشورات والتقارير الرسمية الحالية حول التزام مرضى السل بالعلاج. تهدف الدراسة الحالية إلى الكشف عن مدى الالتزام الفعلي للمرضى حديثي الإصابة بالسل بالعلاج، بالإضافة إلى تحديد العوامل التي تؤدي إلى عدم الالتزام .

المناهج: تشمل الدراسة الأترابية 481 مريضاً من المرضى الذين تم تأكيد إصابتهم حديثاً بالسل وينتمون إلى ثلاث مقاطعات غرب الصين، وقد تم إدراجهم خلال الفترة من شهر يونيو/حزيران حتى شهر ديسمبر/كانون الأول 2012 ومتابعتهم حتى يونيو/حزيران 2013. المرضى الذين فاتتهم جرعة واحدة على الأقل أو لم يقوموا بالمتابعة بإعادة الفحص خلال فترة البرنامج العلاجي يعتبرون غير ملتزمين. تم تحديد العوامل المؤثرة باستخدام نموذج الانحدار اللوجستي.

النتائج: بلغ عدد المرضى غير الملتزمين 173 مريضاً (36.0%)، في حين كان عدد الحالات التي لم تقم بالمتابعة 136 حالة (28.2%). بلغت نسبة المرضى الذين تناولوا العقاقير تحت الإشراف المباشر 13.9%، بينما تم الإشراف على 60.5% من المرضى عبر الاتصال الهاتفي. أشار التحليل العاملي إلى أن المرضى الذين قام أفراد العائلة بالإشراف عليهم (OR:5.54، 95% CI:2.87-10.69) والتي تجاوزت مصاريف الخدمات الشهرية لديهم 450 رنمينبي (OR:2.08، 95% CI:1.35-3.19) هم أكثر احتمالاً للانتماء لغير الملتزمين، بينما كان الإشراف عن طريق الزيارة المنزلية (OR:0.06, 95% CI:0.01-0.28) والاتصال الهاتفي (OR:0.27, 95% CI:0.17-0.44) من العوامل الوقائية.

الاستنتاج: رغم المجهودات التي بُذلت مؤخراً فإن عدداً كبيراً من المرضى الذين تم تأكيد إصابتهم حديثاً بالسل لم يتمكنوا من الالتزام بالعلاج القياسي. هذا إلى جانب أن عدم قيام المرضى بالمتابعة ما زال يمثل مشكلة خطيرة. ضعف الإشراف على العلاج وثقل العبء المالي قد يكونا هما السببين الرئيسيين لعدم الالتزام؛ لذا هناك حاجة إلى القيام بالمزيد لتحسين سياسات الإشراف على العلاج وتقديم الدعم المالي سواءً لمقدمي الخدمة الصحية أو للمرضى.

Translated from English version into Arabic by Ran Abdel Rahman, through

TRANSLATORS
WITHOUT BORDERS

结核病患者能否按医嘱依从治疗？在中国开展的一项前瞻性队列研究结果

雷迅，黄轲，刘琴，杰永凤，汤胜蓝

摘要

引言：结核病患者需要克服诸多困难才能坚持完成漫长而严格的疗程。目前文献及官方报道的结核病患者治疗依从性数据各不相同。本研究旨在了解新发结核病患者实际的治疗依从性，并探讨不依从的主要因素。

方法：将 2012 年 6 月至 12 月期间在中国西部三个县新确诊的 481 名结核病患者纳入前瞻性队列，并随访至 2013 年 6 月。治疗期间漏服一次及以上药物或错过一次及以上定期复查的患者视作不依从。采用 logistic 回归模型进行影响因素分析。

结果：共有 173 名患者（36.0%）没能依从治疗，其中失访人数达 136 人（28.2%）。13.9%的患者在医务人员直接督导下服药，60.5%的患者接受电话提醒督导。因素分析表明，在家人督促下服药（*OR*:5.54，95% *CI*:2.87-10.69）和每月治疗花费在 450 元以上（*OR*:2.08，95% *CI*:1.35-3.19）的患者更有可能不依从，而医

务人员家访督导（*OR*:0.06, 95% CI:0.01-0.28）及电话督导（*OR*:0.27, 95% *CI*:0.17-0.44）是促进依从性的保护因素。

结论：目前仍有大量新发结核病患者不能坚持完成结核病治疗，同时，患者失访仍是一个严重问题。薄弱的治疗监督和沉重的经济负担是造成患者不依从的主要原因。因此，强化治疗督导措施、加强对医生及患者的经济支持是当前工作的重要内容。

Translated from English version into Chinese by Xun Lei.

Les patients atteints de tuberculose suivent-ils les traitements prescrits en Chine ? Résultats d’une étude de cohorte prospective

Xun Lei, Ke Huang, Qin Liu, Yong-Feng Jie, Sheng-Lan Tang

Résumé

Contexte : des patients atteints de tuberculose (TB) font face à de nombreuses difficultés dans le cadre du respect du schéma thérapeutique anti-tuberculeux rigoureux et à long terme. Les observations relatives à l’observance thérapeutique de patients atteints de TB varient parmi la littérature existante et les rapports officiels. La présente étude a pour objectif de déterminer l’observance thérapeutique actuelle de nouveaux patients atteints de TB et d’identifier des facteurs à l’origine de la non-observance.

Méthodes : une cohorte prospective de 481 cas de TB récemment confirmés provenant de trois districts de l’ouest de la Chine a été recrutée de juin à décembre 2012 et a fait l’objet d’un suivi jusque juin 2013. Des patients ayant manqué au moins une dose de médicament ou un examen de suivi au cours du traitement étaient considérés comme des patients ne suivant pas le traitement. Des facteurs influents ont été identifiés à l’aide d’un modèle de régression logistique.

Résultats : au total, 173 patients (36,0 %) présentaient une absence d’observance thérapeutique et le nombre de cas perdus de vue atteignait 136 patients (28,2 %). Seuls 13,9 % des patients prenaient des médicaments sous surveillance directe et 60,5 % des patients faisaient l’objet d’un suivi par téléphone. Les analyses des facteurs ont suggéré que les patients qui étaient observés par des membres de leur famille (OR : 5,54, IC à 95 % : 2,87-10,69) et payaient plus de 450 RMB pour des prestations mensuelles (OR : 2,08, IC à 95 % : 1,35-3,19) étaient plus susceptibles de ne pas respecter le traitement, tandis que les visites à domiciles (OR : 0,06, IC à 95 % : 0,01-0,28) et les appels téléphoniques (OR : 0,27, IC à 95 % :0,17-0,44) constituaient des facteurs de protection.

Conclusions : malgré de récents efforts, une importante proportion de cas de tuberculose récemment confirmés ne pouvaient pas suivre un traitement antituberculeux standard et le nombre de patients perdus de vue continuait à constituer un grave problème. L’existence d’une surveillance thérapeutique insuffisante et le fait que le traitement implique un important fardeau financier peuvent constituer les principales causes de non-observance. Il convient de multiplier les efforts afin d’améliorer les politiques de surveillance thérapeutique ainsi que les aides financières apportées à la fois aux professionnels de santé et aux patients atteints de tuberculose.

Translated from English version into French by eric ragu, through

TRANSLATORS
WITHOUT BORDERS

Придерживаются ли пациенты с туберкулезом предписанного лечения в Китае? Результаты проспективного группового исследования

Сюнь Лэй (Xun Lei), Кэ Хуан (Ke Huang), Цинь Лю (Qin Liu), Юн Фэн Цзю (Yong-Feng Jie), Шэн-Лань Тань (Sheng-Lan Tang)

Выдержка

Вводная информация: пациенты с туберкулезом имеют множество трудностей с соблюдением строгого долгосрочного режима лечения от туберкулеза. В литературных источниках и официальных отчетах приводятся различные результаты исследований касательно соблюдения пациентами режима лечения. Целью настоящего исследования является получение текущих данных относительно соблюдения режима лечения новыми пациентами с туберкулезом и выявление факторов, ведущих к нарушению данного режима.

Методы: в проспективную группу в период с июня по декабрь 2012 года был внесен 481 пациент с недавно выявленным туберкулезом из трех округов западного Китая. Последующее наблюдение велось до июня 2013 года. Пациенты, пропустившие по меньшей мере один прием лекарств или один последующий осмотр в рамках курса лечения, отмечены как не соблюдающие режим лечения. Факторы влияния были выявлены посредством регрессионной логистической модели.

Результаты: общее количество пациентов, не соблюдающих режим лечения, составило 173 человека (36,0 %), а количество пациентов, не явившихся на последующий осмотр, достигло 136 человек (28,2 %). Лишь 13,9 % пациентов принимали лекарственные препараты под непосредственным наблюдением, контроль 60,5 % пациентов осуществлялся в телефонном режиме. Анализ факторов позволяет предположить, что пациенты, лечение которых контролировали члены семьи (ОШ:5,54, 95% ДИ:2,87–10,69) и которые несли ежемесячные расходы на обслуживание свыше 450 юаней (ОШ:2,08, 95% ДИ:1,35–3,19), были более склонны к нарушению режима лечения, в то время как посещение пациентов на дому (ОШ:0,06, 95% ДИ:0,01–0,28) и контроль посредством телефонных звонков (ОШ:0,27, 95% ДИ:0,17–0,44) выступали в роли защитных факторов.

Выводы: несмотря на приложенные в последнее время усилия существенная часть пациентов с недавно выявленным туберкулезом не смогли соблюдать стандартный режим лечения туберкулеза. Неявка пациентов на последующие осмотры также по-прежнему остается серьезной проблемой. Основными причинами нарушения режима лечения может быть некачественный контроль лечения и значительная финансовая нагрузка. Необходимо расширить мероприятия по усовершенствованию принципов контроля процесса лечения и предоставлению финансовой поддержки медицинским учреждениям и пациентам с туберкулезом.

Translated from English version into Russian by Irina Gladkova, through

TRANSLATORS
WITHOUT BORDERS

¿Cumplen los pacientes con tuberculosis en China con los tratamientos prescritos? Resultados de un estudio

de cohorte prospectivo

Xun Lei, Ke Huang, Qin Liu, Yong-Feng Jie, Sheng-Lan Tang

Resumen

Antecedentes: Los pacientes con tuberculosis (TB) se enfrentan a varias dificultades cuando tienen que cumplir con regímenes de tratamiento rigurosos y a largo plazo para la TB. Existen variaciones en los hallazgos en cuanto al cumplimiento del tratamiento en pacientes con TB en la literatura existente y en los informes oficiales. El presente estudio intentó determinar el cumplimiento real del tratamiento en pacientes nuevos con TB e identificar factores que resultan en la falta de cumplimiento.

Métodos: Se inscribió a una cohorte prospectiva de 481 nuevos pacientes confirmados con TB de tres condados occidentales en China entre junio y diciembre de 2012 y se les hizo seguimiento hasta junio del 2013. Se consideró que aquellos pacientes que perdieron por lo menos una dosis de medicamento o faltaron a una consulta de seguimiento durante el curso del tratamiento no cumplieron con el mismo. Se identificaron los factores influyentes mediante un modelo de regresión logística.

Resultados: Un total de 173 pacientes (36,0%) no cumplieron con el tratamiento y los casos que perdieron citas de seguimiento llegaron a 136 (28,2%). Solo 13,9% de los pacientes tomaron medicamentos bajo observación directa y 60,5% de los pacientes fueron supervisados mediante llamadas telefónicas. El análisis factorial sugirió que era más probable que los pacientes que fueron observados por miembros de sus familias (O:5,54, 95% IC:2,87-10,69) y que pagaban gastos mensuales de servicio por arriba de 450 RMB (O:2,08, 95% IC:1,35-3,19) no cumplieran con el tratamiento, mientras que la supervisión mediante visitas domiciliarias (O:0,06, 95% IC:0,01-0,28) y llamadas telefónicas (O:0,27, 95% IC:0,17-0,44) fueron factores protectores.

Conclusiones: A pesar de esfuerzos recientes, una gran proporción de los pacientes recientemente confirmados con TB no pudieron cumplir con el tratamiento estándar para la TB y la no presentación a consultas de seguimiento continúa siendo un problema serio. La supervisión del tratamiento inadecuada y la gran carga financiera pueden ser las causas principales de la falta de cumplimiento. Se necesita hacer más para mejorar las políticas de supervisión de tratamiento y el apoyo económico tanto a proveedores de salud como a pacientes con TB.

Translated from English version into Spanish by Maria Alejandra Aguada, through

TRANSLATORS WITHOUT BORDERS
